# K_2_V_2_O_2_(AsO_4_)_2_

**DOI:** 10.1107/S1600536812027183

**Published:** 2012-06-23

**Authors:** Sabrina Belkhiri, Djillali Mezaoui, Thierry Roisnel

**Affiliations:** a Laboratoire Sciences des Matériaux, Faculté de Chimie, Université des Sciences et de la Technologie Houari Boumedienne, BP 32 El-Alia 16111 Bab-Ezzouar Alger, Algeria; b CDIFX, UMR 6226, Université de Rennes1, CNRS, Avenue du Général Leclerc, 35042 Rennes Cedex, France

## Abstract

The vanadium oxide arsenate with formula K_2_V_2_O_2_(AsO_4_)_2_, dipotassium divanadium(IV) dioxide diarsenate, has been synthesized by solid-state reaction in an evacuated silica ampoule. Its structure is isotypic with K_2_V_2_O_2_(PO_4_)_2_. The framework is built up from corner-sharing VO_6_ octa­hedra and AsO_4_ tetra­hedra, creating an infinite [VAsO_8_]_∞_ chain running along the *a*- and *c*-axis directions. The K^+^ cations are located in hexa­gonal tunnels, which are delimited by the connection of the [VAsO_8_]_∞_ chains.

## Related literature

For the properties of the potassium titanyl phosphate KTiOPO_4_ (KTP) family, see: El Haidouri *et al.* (1990 ▶); Harrison & Phillips (1999 ▶); Phillips *et al.* (1990 ▶). For the structures of *AM*O*X*O_4_ compounds (*A* = K, Na, Li, *M* = transition metal and *X* = P, As) of the KTP family, see: Phillips *et al.* (1990 ▶); Harrison & Phillips (1999 ▶); El Haidouri *et al.* (1990 ▶). For the synthesis of K_1.65_V_1.78_W_0.22_O_2_(AsO_4_)_2_, see: Belkhiri *et al.* (2009 ▶). For the synthesis and structure of isotypic K_2_V_2_O_2_(PO_4_)_2_, see: Benhamada *et al.* (1991 ▶). For the effect on the electron transport properties caused by the distortion of the V^IV^O_6_ octa­hedra, see: El Haidouri *et al.* (1990 ▶); El Brahimi & Durand (1986 ▶); Nakagawa *et al.* (1999 ▶). For the hexa­gonal tungsten bronze structure, see: Magnéli (1953 ▶).

## Experimental

### 

#### Crystal data

K_2_V_2_O_2_(AsO_4_)_2_
*M* *_r_* = 489.9
Orthorhombic, 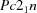

*a* = 6.5368 (2) Å
*b* = 10.7228 (5) Å
*c* = 13.0666 (4) Å
*V* = 915.87 (6) Å^3^
*Z* = 4
Mo *K*α radiation
μ = 10.16 mm^−1^
*T* = 150 K
0.30 × 0.25 × 0.22 mm

#### Data collection

Bruker APEXII diffractometer
Absorption correction: multi-scan (*SADABS*; Sheldrick, 2002 ▶) *T*_min_ = 0.073, *T*_max_ = 0.107
11866 measured reflections
4169 independent reflections
3805 reflections with *I* > 3σ(*I*)
*R*_int_ = 0.033

#### Refinement

*R*[*F* ^2^ > 2σ(*F* ^2^)] = 0.028
*wR*(*F* ^2^) = 0.034
*S* = 1.60
4169 reflections
139 parameters
Δρ_max_ = 0.94 e Å^−3^
Δρ_min_ = −0.77 e Å^−3^
Absolute structure: Flack (1983 ▶), 1256 Friedel pairs
Flack parameter: 0.387 (8)

### 

Data collection: *APEX2* (Bruker, 2004 ▶); cell refinement: *SAINT* (Bruker, 2004 ▶); data reduction: *SAINT*; program(s) used to solve structure: *SuperFlip* (Palatinus & Chapuis, 2007 ▶); program(s) used to refine structure: *JANA2006* (Petříček *et al.*, 2006 ▶); molecular graphics: *ATOMS* (Dowty, 1994 ▶) and *GRETEP* (Laugier & Bochu, 2002 ▶); software used to prepare material for publication: *JANA2006*.

## Supplementary Material

Crystal structure: contains datablock(s) global, I. DOI: 10.1107/S1600536812027183/ru2035sup1.cif

Structure factors: contains datablock(s) I. DOI: 10.1107/S1600536812027183/ru2035Isup2.hkl

Additional supplementary materials:  crystallographic information; 3D view; checkCIF report

## Figures and Tables

**Fig. 1.**
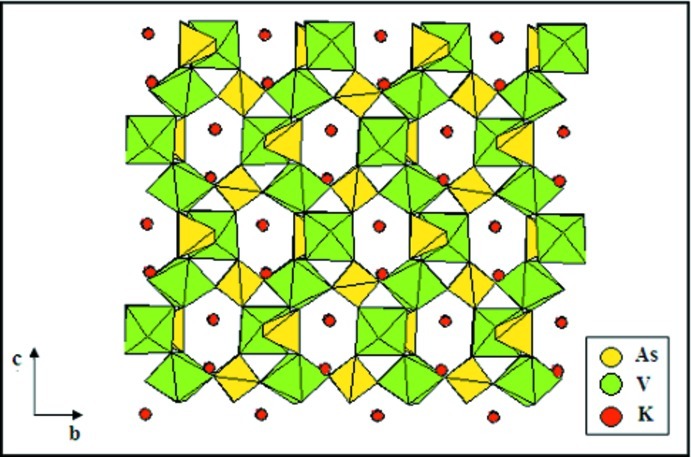
The projection of K2V2O2(AsO4)2 along a.

**Fig. 2.**
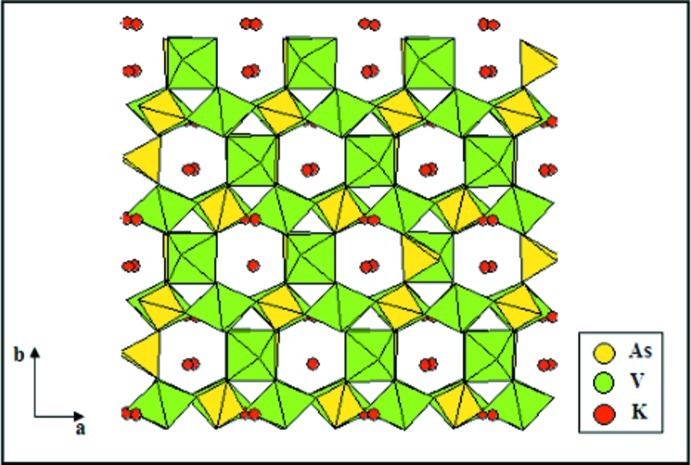
The projection of K2V2O2(AsO4)2 along c.

**Fig. 3.**
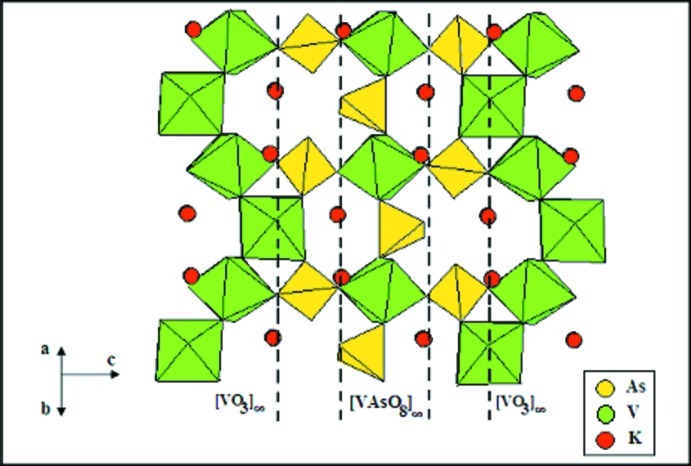
The projection of K2V2O2(AsO4)2 along (011).

**Fig. 4.**
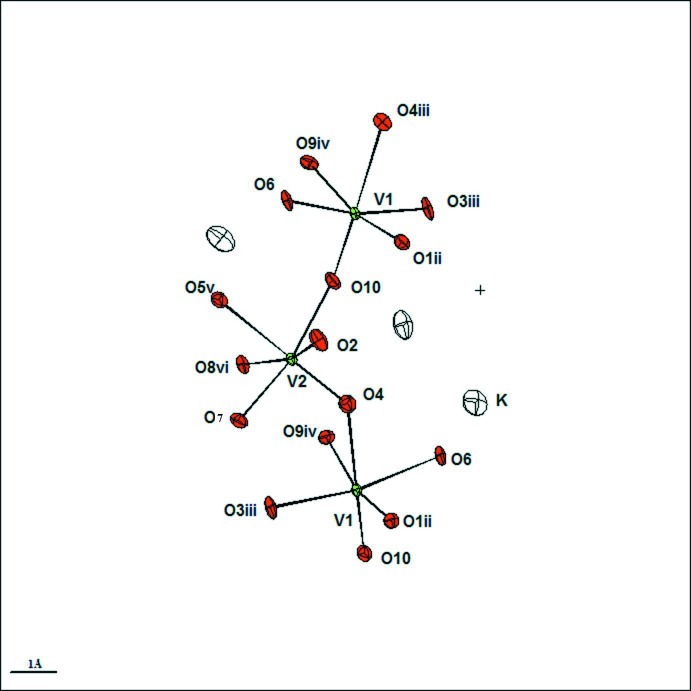
octahedral infinite [VO3]∞ chains running along [011] direction with [Symmetry codes: (i) ?x + 1/2, y, z?1/2; (ii) x + 1, y, z; (iii) ?x + 1/2, y, z + 1/2; (iv) ?x + 1, y + 1/2, ?z; (v) ?x + 1, y?1/2, ?z; (vi) x?1, y, z].

**Fig. 5.**
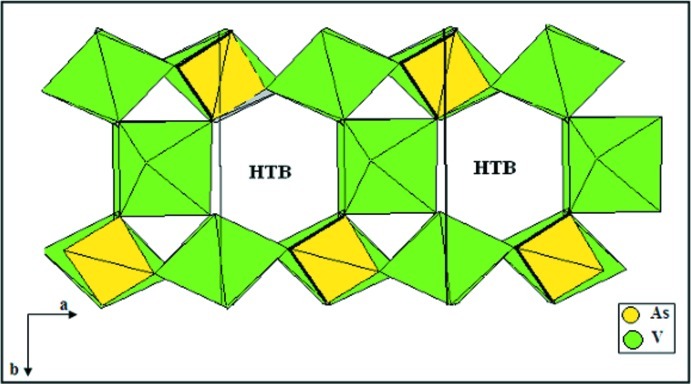
The comparison between K2V2O2(AsO4)2 and HTB structures.

**Fig. 6.**
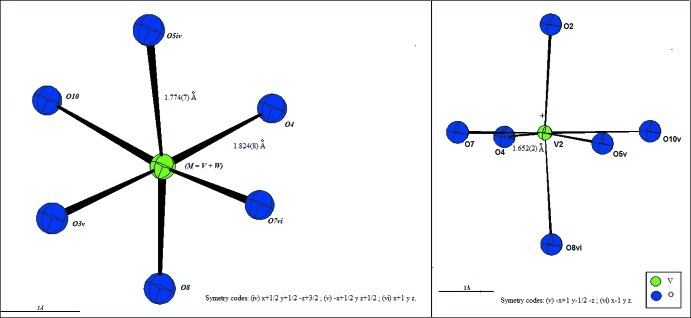
MO6 and VO6 octahedra of K1.65V1.78W0.22O2(AsO4)2 and K2V2O2(AsO4)2 respectively.

## supplementary crystallographic information

### Comment

The compounds belonging to the potassium titanyl phosphate KTiOPO_4_ (KTP) family have been widely studied for their outstanding non linear optical property (NLO)(Phillips *et al.*, 1990; Harrison *et al.*, 1999; El Haidouri *et al.*, 1990).

These materials are characterized by their high laser damage threshold, high electrooptic coefficient and an excellent thermal stability (Phillips *et al.*, 1990; Harrison *et al.*, 1999; El Haidouri *et al.*, 1990)).

The KTP compound is generaly used in a laser system such as second harmonic generation (SGH) for doubling laser light for example in the Nd Yag laser equipement.

The framework of AMOXO_4_ (A: K, Na, Li..), (*M*: tansition metal) and (*X*: P, As) is built up from MO_6_ octahedra and AsO_4_ tetrahedra sharing their corners.

The structure of AMOXO_4_ of KTP family shows an irregular octahedra MO_6_ with one short bond (1.653 (5) Å to 1.851 (5) Å)) (Phillips *et al.*, 1990; Harrison *et al.*, 1999; El Haidouri *et al.*, 1990). In two years later we synthetized a new compound K_1.65_V_1.78_W_0.22_O_2_(AsO_4_)_2_ (Belkhiri *et al.*, 2009) of KTP family, It presents an irregular MO_6_ octahedra with (*M*=V+W). The MO_6_ polyhedra consist of two abnormal short bonds M—O (1.774 (7) Å) and(1.824 (8) Å) which suggest that the NLO property could be more important in this compound, because the most physical related to structural studies showed that the non linear optical property is due to the short bond in the octahedral polyhedra.

We are interested on K_1.65_V_1.78_W_0.22_O_2_(AsO_4_)_2_ for these two short bonds, we substituted the tungsten by the vanadium element in order to show the influence of the tungsten and vanadium on the distortion of the MO_6_ octahedra.

We synthetized and studied the structure of new single-crystal K_2_V_2_O_2_(AsO_4_)_2_ isotype to KVOPO_4_ (Benhamada *et al.*, 1991), we describe here the structure of K_2_V_2_O_2_(AsO_4_)_2_.

The framework [VAsO_5_]_∞_ is built up from single VO_6_ octahedra sharing corners with AsO_4_ tetrahedra. The projection of K_2_V_2_O_2_(AsO_4_)_2_ structure along *a* and *c* directions (Fig. 1) and (Fig. 2) respectively shows the existence of infinite [VAsO_8_]_∞_ chains running along *a* and *c* directions.

Two infinite [VAsO_8_]_∞_ chains oriented along *a* are linked *via* infinite [VAsO_8_]_∞_ chains running along *c* and *vice versa*. The AsO_4_ tetrahedra share their corners with four VO_6_ octahedra, and the VO_6_ octahedra share their corners with four AsO_4_ tetrahedra and two VO_6_ octahedra.

This arrangement creates an octahedral infinite [VO_3_]_∞_ chains running along [011] direction (Fig. 3) and (Fig. 4). The existence of one dimensional octahedral infinite [VO_3_]_∞_ chains pretends the possibility of electronic transport properties. These properties can be accentuated by the distortion of the octahedral polyhedra occupied by V(IV) with the d1 configuration (El Haidouri *et al.*, 1990; Nakagawa *et al.*, 1999; El Brahimi & Durand, 1986).

The framework [VAsO_5_]_∞_ delimits two sorts of hexagonal tunnels running along *a* and *c* directions, where the potassium ions are located. The first tunnel (Fig. 1) results from junction of three VO_6_ octahedra and three AsO_4_ tetrahedra linked by their corners. Whereas the second type of tunnel (Fig. 2) is formed of rings of four VO_6_ octahedra and two AsO_4_ tetrahedra linked by their corners.

The great similarity of the framework [VAsO_5_]_∞_ with hexagonal tungsten bronze structure (HTB) described by Magnéli (1953) concerns necessary the hexagonal tunnel which can be deduced from HTB tunnels by replacing two octahedra out of six in an ordered way by two AsO_4_ tetrahedra (Fig. 5).

As a result, the remplacement of tungsten by the vanadium element in the structure of K_1.65_V_1.78_W_0.22_O_2_(AsO_4_)_2_ led to irregular octahedra VO_6_ with one short (1.6551 (18) Å) and one long bond V—O (2.2301 (18) Å). Whereas in the case of K_1.65_V_1.78_W_0.22_O_2_(AsO_4_)_2_, the presence of the tungsten element in MO_6_ with (*M*=V+W) creates an irregular octahedra MO_6_ with two short bonds M—O (1.774 (7) Å) and(1.824 (8) Å).

The comparaison between the MO_6_ octahedra of K_1.65_V_1.78_W_0.22_O_2_(AsO_4_)_2_ and VO_6_ octahedra of K_2_V_2_O_2_(AsO_4_)_2_ (Fig. 6) shows clearly that the distortion is more important in MO_6_ than in VO_6_ due to the mixed occupation of the MO_6_ by the tungsten and vanadium simultaneously. Furthermore the mixed occupation creates two short bonds which can have an impact on the non linear optical property.

Among our perspectives is to realise the non linear optical property for K_1.65_V_1.78_W_0.22_O_2_(AsO_4_)_2_ and K_2_V_2_O_2_(AsO_4_)_2_ and to show the influence of the vanadium and the tungsten on the second harmonic generation.

### Experimental

The growth of the single-crystal K_2_V_2_O_2_(AsO_4_)_2_ was performed in two steps: firstly, the stoechiometric mixture of K_2_CO_3_, V_2_O_5_, and As_2_O_5_ was heated in platinium crucible for 24 h at 573 K in order to decompose the potassium carbonate. Secondly the appropriate amount of vanadium was added into the mixture and then heated at 973 K for 7 days in evacuated silica ampoule. From the resulting mixture some dark single crystals were extracted.

### Refinement

Refinement of *F*^2^ against ALL reflections. The weighted *R*-factor *wR* and goodness of fit *S* are based on *F*^2^, conventional *R*-factors are based on *F*, with *F* set to zero for negative *F*^2^. The threshold expression of *F*^2^ > n*σ(*F*^2^) is used only for calculating *R*-factors *etc*. and is not relevant to the choice of reflections for refinement.

### Crystal data

**Table:**

K_2_V_2_O_2_(AsO_4_)_2_	*F*(000) = 920
*M**_r_* = 489.9	*D*_x_ = 3.552 Mg m^−^^3^
Orthorhombic, *P**c*2_1_*n*	Mo *K*α radiation, λ = 0.71069 Å
Hall symbol: P -2n -2ac	Cell parameters from 5952 reflections
*a* = 6.5368 (2) Å	θ = 3.5–40.2°
*b* = 10.7228 (5) Å	µ = 10.16 mm^−^^1^
*c* = 13.0666 (4) Å	*T* = 150 K
*V* = 915.87 (6) Å^3^	Prism, black
*Z* = 4	0.3 × 0.25 × 0.22 mm

### Data collection

**Table:**

Bruker APEXII diffractometer	4169 independent reflections
Radiation source: fine-focus sealed tube	3805 reflections with *I* > 3σ(*I*)
Graphite monochromator	*R*_int_ = 0.033
CCD rotation images, thin slices scans	θ_max_ = 40.2°, θ_min_ = 3.5°
Absorption correction: multi-scan (*SADABS*; Sheldrick, 2002)	*h* = −23→23
*T*_min_ = 0.073, *T*_max_ = 0.107	*k* = −11→10
11866 measured reflections	*l* = −19→15

### Refinement

**Table:**

Refinement on *F*	Weighting scheme based on measured s.u.'s *w* = 1/(σ^2^(*F*) + 0.000049*F*^2^)
*R*[*F*^2^ > 2σ(*F*^2^)] = 0.028	(Δ/σ)_max_ = 0.003
*wR*(*F*^2^) = 0.034	Δρ_max_ = 0.94 e Å^−^^3^
*S* = 1.60	Δρ_min_ = −0.77 e Å^−^^3^
4169 reflections	Extinction correction: B-C type 1 Gaussian isotropic (Becker & Coppens, 1974)
139 parameters	Extinction coefficient: 970 (110)
0 restraints	Absolute structure: Flack (1983), 1256 Friedel pairs
0 constraints	Flack parameter: 0.387 (8)

### Fractional atomic coordinates and isotropic or equivalent isotropic displacement parameters (Å^2^)

**Table:**

	*x*	*y*	*z*	*U*_iso_*/*U*_eq_	
As2	0.99887 (4)	0.333103	−0.18131 (2)	0.00293 (5)	
V1	0.27644 (5)	0.59847 (6)	0.25107 (3)	0.00255 (8)	
V2	0.49984 (6)	0.34895 (5)	−0.12277 (3)	0.00271 (8)	
K1	−0.22511 (9)	0.53150 (8)	0.12103 (6)	0.01172 (15)	
K2	0.79227 (10)	0.77688 (8)	−0.10496 (5)	0.01127 (15)	
O1	−0.5484 (3)	0.4499 (2)	0.23637 (18)	0.0058 (5)	
O2	0.7996 (3)	0.3653 (2)	−0.10470 (17)	0.0060 (3)	
O3	0.1652 (3)	0.6001 (3)	−0.09922 (15)	0.0060 (4)	
O4	0.4565 (3)	0.4521 (2)	−0.21415 (18)	0.0060 (5)	
O5	0.4859 (3)	0.7051 (2)	0.01550 (19)	0.0061 (5)	
O6	0.1747 (3)	0.5671 (2)	0.10771 (16)	0.0053 (4)	
O7	0.4687 (4)	0.4564 (2)	−0.00520 (17)	0.0063 (5)	
O8	1.2015 (3)	0.2971 (2)	−0.10749 (17)	0.0060 (3)	
O9	0.9597 (3)	0.2103 (2)	−0.26290 (18)	0.0062 (5)	
O10	0.4516 (3)	0.7042 (2)	0.22272 (18)	0.0061 (5)	
As1	0.32206 (3)	0.58763 (5)	0.00215 (2)	0.00258 (5)	

### Atomic displacement parameters (Å^2^)

**Table:**

	*U*^11^	*U*^22^	*U*^33^	*U*^12^	*U*^13^	*U*^23^
As2	0.00216 (8)	0.00404 (10)	0.00258 (10)	−0.00032 (7)	−0.00002 (9)	0.00035 (10)
V1	0.00278 (13)	0.00322 (15)	0.00164 (15)	0.00008 (15)	0.00028 (13)	0.00010 (14)
V2	0.00261 (13)	0.00336 (17)	0.00216 (15)	0.00019 (13)	−0.00004 (13)	0.00031 (14)
K1	0.0052 (2)	0.0156 (3)	0.0144 (3)	−0.0015 (2)	−0.0016 (2)	0.0047 (3)
K2	0.0104 (2)	0.0171 (3)	0.0063 (2)	−0.0012 (2)	0.00160 (19)	−0.0001 (3)
O1	0.0065 (7)	0.0053 (8)	0.0057 (9)	0.0026 (6)	0.0020 (7)	0.0030 (7)
O2	0.0028 (4)	0.0093 (5)	0.0060 (5)	−0.0001 (4)	−0.0006 (5)	0.0005 (6)
O3	0.0046 (6)	0.0114 (9)	0.0020 (7)	0.0014 (7)	−0.0015 (5)	0.0004 (8)
O4	0.0046 (7)	0.0073 (8)	0.0063 (9)	0.0009 (6)	−0.0009 (6)	0.0011 (7)
O5	0.0062 (8)	0.0071 (8)	0.0049 (9)	−0.0031 (7)	0.0010 (6)	−0.0017 (7)
O6	0.0044 (7)	0.0088 (9)	0.0026 (7)	0.0013 (6)	0.0005 (6)	0.0012 (7)
O7	0.0069 (8)	0.0079 (8)	0.0043 (9)	0.0035 (7)	−0.0020 (6)	−0.0017 (7)
O8	0.0028 (4)	0.0093 (5)	0.0060 (5)	−0.0001 (4)	−0.0006 (5)	0.0005 (6)
O9	0.0066 (7)	0.0067 (8)	0.0052 (9)	−0.0025 (7)	0.0018 (7)	−0.0018 (8)
O10	0.0075 (8)	0.0070 (8)	0.0039 (8)	−0.0012 (7)	0.0011 (7)	0.0012 (7)
As1	0.00244 (8)	0.00385 (9)	0.00144 (9)	−0.00024 (9)	−0.00003 (8)	−0.00044 (9)

### Geometric parameters (Å, º)

**Table:**

As2—O1^i^	1.682 (2)	V2—O2	1.9811 (19)
As2—O2	1.679 (2)	V2—O4	1.652 (2)
As2—O8	1.684 (2)	V2—O5^v^	2.087 (2)
As2—O9	1.714 (2)	V2—O7	1.931 (2)
V1—O1^ii^	1.971 (2)	V2—O8^vi^	2.0375 (19)
V1—O3^iii^	1.993 (2)	V2—O10^v^	2.053 (2)
V1—O4^iii^	2.234 (2)	O3—As1	1.6805 (19)
V1—O6	2.016 (2)	O5—As1	1.662 (2)
V1—O9^iv^	1.960 (2)	O6—As1	1.697 (2)
V1—O10	1.654 (2)	O7—As1	1.705 (2)
			
O1^i^—As2—O2	112.20 (11)	O4—V2—O5^v^	171.20 (10)
O1^i^—As2—O8	112.68 (10)	O4—V2—O7	99.06 (11)
O1^i^—As2—O9	101.71 (11)	O4—V2—O8^vi^	95.14 (10)
O2—As2—O8	108.44 (10)	O4—V2—O10^v^	94.17 (10)
O2—As2—O9	114.38 (10)	O5^v^—V2—O7	84.92 (10)
O8—As2—O9	107.32 (11)	O5^v^—V2—O8^vi^	77.00 (9)
O1^ii^—V1—O3^iii^	89.52 (10)	O5^v^—V2—O10^v^	82.04 (9)
O1^ii^—V1—O4^iii^	81.24 (9)	O7—V2—O8^vi^	89.08 (10)
O1^ii^—V1—O6	88.06 (9)	O7—V2—O10^v^	166.76 (10)
O1^ii^—V1—O9^iv^	163.57 (9)	O8^vi^—V2—O10^v^	90.22 (9)
O1^ii^—V1—O10	97.49 (10)	As2^iii^—O1—V1^vi^	130.63 (13)
O3^iii^—V1—O4^iii^	86.39 (9)	As2—O2—V2	132.71 (13)
O3^iii^—V1—O6	167.57 (9)	V1^i^—O3—As1	131.00 (10)
O3^iii^—V1—O9^iv^	93.90 (10)	V1^i^—O4—V2	137.25 (13)
O3^iii^—V1—O10	94.66 (11)	V2^iv^—O5—As1	131.25 (13)
O4^iii^—V1—O6	81.19 (9)	V1—O6—As1	123.11 (10)
O4^iii^—V1—O9^iv^	82.94 (9)	V2—O7—As1	126.62 (13)
O4^iii^—V1—O10	178.35 (10)	As2—O8—V2^ii^	129.37 (13)
O6—V1—O9^iv^	85.16 (9)	As2—O9—V1^v^	122.59 (12)
O6—V1—O10	97.74 (10)	V1—O10—V2^iv^	140.17 (13)
O9^iv^—V1—O10	98.24 (11)	O3—As1—O5	114.55 (12)
O2—V2—O4	101.33 (10)	O3—As1—O6	107.73 (9)
O2—V2—O5^v^	86.62 (9)	O3—As1—O7	111.36 (12)
O2—V2—O7	87.53 (10)	O5—As1—O6	112.26 (11)
O2—V2—O8^vi^	163.51 (9)	O5—As1—O7	105.60 (11)
O2—V2—O10^v^	89.41 (9)	O6—As1—O7	104.94 (11)

Symmetry codes: (i) −*x*+1/2, *y*, *z*−1/2; (ii) *x*+1, *y*, *z*; (iii) −*x*+1/2, *y*, *z*+1/2; (iv) −*x*+1, *y*+1/2, −*z*; (v) −*x*+1, *y*−1/2, −*z*; (vi) *x*−1, *y*, *z*.
